# *In Vitro *inhibitory activity of *Alpinia katsumadai *extracts against influenza virus infection and hemagglutination

**DOI:** 10.1186/1743-422X-7-307

**Published:** 2010-11-10

**Authors:** Hyung-Jun Kwon, Ha-Hyun Kim, So Young Yoon, Young Bae Ryu, Jong Sun Chang, Kyoung-Oh Cho, Mun-Chual Rho, Su-Jin Park, Woo Song Lee

**Affiliations:** 1 Eco-Friendly Biomaterial Research Center and AI Control Material Research Center, Korea Research Institute of Bioscience and Biotechnology, Jeongeup 580-185, Republic of Korea; 2 Biotherapy Human Resources Center, College of Veterinary Medicine, Chonnam National University, Gwangju 500-757, Republic of Korea

## Abstract

**Background:**

*Alpinia katsumadai *(AK) extracts and fractions were tested for *in vitro *antiviral activities against influenza virus type A, specially human A/PR/8/34 (H1N1) and avian A/Chicken/Korea/MS96/96 (H9N2), by means of time-of-addition experiments; pre-treatment, simultaneous treatment, and post treatment.

**Results:**

In pre-treatment assay, the AK extracts and AK fractions did not show significant antiviral activity. During the simultaneous treatment assay, one AK extract and five AK fractions designated as AK-1 to AK-3, AK-5, AK-10, and AK-11 showed complete inhibition of virus infectivity against A/PR/8/34 (H1N1) and A/Chicken/Korea/MS96/96 (H9N2). The 50% effective inhibitory concentrations (EC_50_) of these one AK extracts and five AK fractions with exception of the AK-9 were from 0.8 ± 1.4 to 16.4 ± 4.5 *μ*g/mL against A/PR/8/34 (H1N1). The two AK extracts and three AK fractions had EC_50 _values ranging from <0.39 ± 0.4 to 2.3 ± 3.6 *μ*g/mL against A/Chicken/Korea/MS96/96 (H9N2). By the hemagglutination inhibition (HI) assay, the two AK extracts and five AK fractions completely inhibited viral adsorption onto chicken RBCs at less than 100 *μ*g/mL against both A/PR/8/34 (H1N1) and A/Chicken/Korea/MS96/96 (H9N2). Interestingly, only AK-3 was found with inhibition for both viral attachment and viral replication after showing extended antiviral activity during the post treatment assay and quantitative real-time PCR.

**Conclusions:**

These results suggest that AK extracts and fractions had strong anti-influenza virus activity that can inhibit viral attachment and/or viral replication, and may be used as viral prophylaxis.

## Background

Influenza viruses are enveloped RNA viruses that belong to the family *Orthomyxoviridae*, including influenza viruses A, B, and C, and two other genera [1,2]. The viruses are responsible for seasonal flu epidemic and caused acute contagious respiratory infection. Particularly, young children, the old, and patients with chronic diseases are at high risk to develop severe complications of influenza virus infection that lead to high mortality rates [3,4]. Among the five genera, type A viruses are the most virulent human pathogen which have caused three pandemics in the 20^th ^century and are known to be transmitted to other species [1,2]. More recently, the human influenza outbreak of the swine-origin A/H1N1 strain in 2009 has become a serious public concern around the world [5,6].

Up to present, there are only four antiviral agents, approved by the FDA to treat influenza virus infection and these can be divided into two groups. The group comprising of amantadine and rimantadine block the M2 ion channel, which is essential for viral proliferation, thereby interfering with viral uncoating inside cells. The group of zanamivir and oseltamivir inhibit viral neuraminidase which plays an important role in viral release [1,7]. The M2 inhibitors are effective only against influenza virus A and are associated with several toxic effects in the digestive and autonomic nervous systems, as well as with the emergence of drug-resistant variants throughout the 40 years of its use [8]. Although zanamivir and oseltamivir have high antiviral activity, the bioavailability of zanamivir is low and it is excreted rapidly by the kidneys. Nausea and vomiting are frequent among adults receiving oseltamivir [9,10]. Besides these two major groups of anti-influenza drugs, several other approaches including inhibition of viral RNA transcription (RNA polymerase), small interfering RNA, inhibition of virus-cell fusion and proteolytic processing of hemagglutinin (HA) are existing; however, all of these alternative methods has not yet been licensed [7,11]. Hence, the need for a new drug and their market are greatly emphasized.

*Alpinia katsumadai *Hayata (Zingiberaceae) (AK) has been utilized as a traditional Chinese herbal drug for an anti-emetic and stomachic [12]. It has been reported to contain a variety of diarylheptanoids, monoterpenes, sesquiterpenoid, flavonoids, and chalcones as major constituents [13-15]. Recently, compounds isolated from *A. katsumadai *showed *in vitro *neuraminidase inhibitory activities against human influenza virus A/PR/8/34 of subtype H1N1 and antiviral effects in plaque reduction assays of the four H1N1 swine influenza viruses [16]. However, the antiviral mechanisms of AK is not clear. Therefore, in this study, we investigated the *in vitro *anti-influenza viral mechanism of AK extracts and AK fractions using time-of-addition and hemagglutination inhibition (HI) assays.

## Methods

### Preparation of *Alpinia katsumadai *extracts and fractions

The dried seeds (4.8 kg) of AK were ground and macerated with ethanol (1.5 L × 20) for one week at room temperature, and then filtered and the clarified solvent was evaporated under reduced pressure to afford the ethanol extract (289 g, AK-1). The combined ethanol extract was dissolved in 2.0 L of a mixture of water and ethanol (1:9) and successively partitioned with EtOAc and water, yielding an EtOAc fraction (192 g, AK-2) and water fraction (70 g, AK-3). Then, the water soluble fraction AK-3 was subjected to diaion (HP-20) column chromatography, eluted with MeOH in water in a step-gradient manner from 20% to 100% to make five fractions [20% methanol (AK-4): 3.9 g, 40% methanol (AK-5): 11.9 g, 60% methanol (AK-6): 32.7 g, 80% methanol (AK-7): 3.8 g, and 100% methanol (AK-8): 1.1 g]. To obtain polysaccharide fraction, we reexamined another procedure. The dried and pulverized seeds of AK (600 g) were mixed with 1.5 L of water and shaken at 80°C for 12 h. The water extract (98 g, AK-9) was filtered through a filter paper to remove debris, and then the solution was precipitated by the addition of ethanol in 1:4 ratio (v/v) at room temperature. After overnight precipitation, the precipitate was collected by centrifugation (12,000 rpm, 30 min at 4°C) and washed with acetone and freeze-dried. This fractionation procedure was repeated three times. The corresponding fraction (56 g, AK-10) was light brown powder (polysaccharide fraction). The remained supernatant was concentrated in a rotary evaporator under reduced pressure, yielding a supernatant fraction (28 g, AK-11).

### Cells and viruses

Madin-Darby canine kidney (MDCK) cells were obtained from the American Type Culture Collection (ATCC CCL-3; Manassas, VA, USA) and grown in Eagle's minimum essential medium (EMEM) supplemented with 10% fetal bovine serum (FBS), 100 U/mL penicillin, and 100 *μ*g/mL streptomycin. The influenza strains A/PR/8/34 (H1N1) (ATCC VR-1469) and A/Chicken/Korea/MS96/96 (H9N2) were propagated in MDCK cells in the presence of 10 *μ*g/mL trypsin (1:250; GIBCO Invitrogen Corporation, California).

### Cytotoxicity

MDCK cells were grown in 96 well plates at 1 × 10^5 ^cells/well for 24 h. The media in plates were replaced with media containing serially diluted extracts and incubated for 72 h. The solution was replaced with only media and 5 *μ*L MTT (3-[4,5-dimethylthiozol-2-yl]-2,5-diphenyltetrazolium bromide; Sigma, St. Louis, MO) solution was added to each well and incubated at 37°C for 4 h. The supernatant was removed, and 100 *μ*L 0.04 M HCl-isopropanol was added to dissolve formazan crystals. Absorbance was measured at 540 nm with subtraction of the background measurement at 655 nm in a microplate reader. The 50% cytotoxic concentration (CC_50_) was calculated by regression analysis.

### Antiviral assay

**Figure 1:**
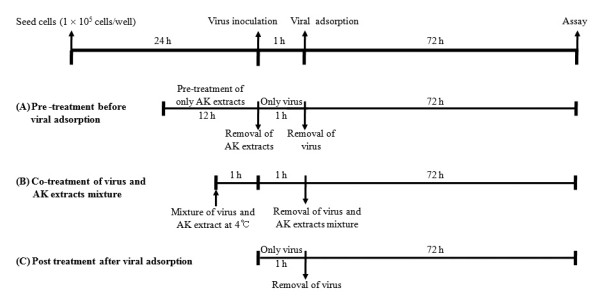
**Antiviral assay strategies with AK extracts and AK fractions on different stages of virus infection**. At 12 h prior to virus infection for pre-treatment assay (**A**), at the same time after virus incubation with AK extracts and AK fractions at 4°C for 1 h for simultaneous treatment assay (**B**), and at 1 h later of AK extracts after viral infection for post treatment assay (**C**).

Pre-treatment assay (Figure 1A): MDCK cells were grown in 96 well plates at 1 × 10^5 ^cells/well for 24 h. Before virus inoculation, non cytotoxic concentration (≤ CC_50_) of AK extracts were added to the cells and incubated for 12 h. Then two AK extracts and five AK fractions were removed and the MDCK cells were washed 2 times with PBS. Influenza virus at 100 TCID_50 _(tissue culture infectious dose) were inoculated onto the MDCK cells for 1 h with occasional rocking. The virus was removed and the cells replaced with EMEM containing 10 *μ*g/mL trypsin. The cultures were incubated for 72 h at 35°C under 5% CO_2 _atmosphere until the cells in the infected, untreated control well showed complete viral cytopathic effect (CPE) as observed by light microscopy. Each concentration of two AK extracts and five AK fractions was assayed in triplicate.

Simultaneous treatment assay (Figure 1B): Various concentrations of two AK extracts and five AK fractions were mixed with virus and incubated at 4°C for 1 h. The mixture were inoculated onto near confluent MDCK cell monolayers (1 × 10^5 ^cells/well) for 1 h with occasional rocking. The solution was removed and the media was replaced with EMEM containing 10 *μ*g/mL trypsin. The cultures were incubated for 72 h at 35°C under 5% CO_2 _atmosphere until the cells in the infected, untreated control well showed complete CPE as observed by light microscopy. Each concentration of two AK extracts and five AK fractions was assayed in triplicate.

Post treatment assay (Figure 1C): Influenza virus at 100 TCID_50 _were inoculated onto near confluent MDCK cell monolayers (1 × 10^5 ^cells/well) for 1 h with occasional rocking. The media was removed and replaced by EMEM containing 10 *μ*g/mL trypsin and several two AK extracts and five AK fractions at different concentrations. The cultures were incubated for 72 h at 35°C under 5% CO_2 _atmosphere until the cells in the infected, untreated control well showed complete viral CPE as observed by light microscopy. The two AK extracts and five AK fractions were assayed for virus inhibition in triplicate.

After 72 h incubation in all antiviral assays, 0.034% neutral red was added to each well and incubated for 2 h at 35°C in the dark. The neutral red solution was removed and the cells were washed with PBS (pH 7.4). Destaining solution (containing 1% glacial acetic acid, 49% H_2_O, and 50% ethanol) was added to each well. The plates were incubated in the dark for 15 min at room temperature. Absorbance was read at 540 nm using a microplate reader.

### Hemagglutination inhibition (HI) assay

The hemagglutination inhibition assay was performed to evaluate the effects of two AK extracts and five AK fractions on viral adsorption to target cells. Standardized chicken red blood cell (cRBC) solutions were prepared according to the WHO manual 2002 (WHO, 2002). The influenza virus solution (4 HAU/25 *μ*L) was mixed with an equal volume of AK extracts (25 *μ*L) in a two-fold serial dilution in PBS (pH 7.4) for 1 h at 4°C. Fifty *μ*L of the solution was mixed with an equal volume of a 1% cRBC suspension and incubated for 1 h at room temperature.

### Reverse Transcription and quantitative real-time PCR

MDCK cells were grown to about 90% confluence, infected with influenza virus at 0.01 MOI, and cultured in the presence of AK-3 (20 *μ*g/mL) or tamiflu (10 *μ*M). Medium was removed after 3 h and 18 h. Cells were scraped off, washed twice with PBS, and collected by centrifugation (500 g for 3 min). In order to determine the expression level of Matrix (M) gene mRNA of influenza virus, total RNA was isolated using Qiagen RNeasy mini kit (QIAGEN) according to manufacturer's instruction. The primer sequences used for quantitative real-time PCR of viral RNA were 5'-CTTCTAACCGAGGTCGAAACGTA-3' (sense) and 5'- GGTGACAGGATTGGTCTTGTCTTTA-3' (antisense) [17]. The GAPDH was used as internal control of cellular RNAs, with primer sequences of 5'-CAACGGATTTGGCCGTATTGG-3' (sense) and Reverse: 5'- TGAAGGGGTCATTGATGGCG-3 (antisense).

The total RNA was reverse transcribed into cDNA using the High Capacity RNA-to-cDNA master mix (Applied Biosystems) according to the manufacturer's protocol. Reverse transcription was performed at 42°C for 1 h. The enzyme was inactivated at 95°C for 5 min. The cDNA was stored at -20°C or directly used in quantitative real-time PCR. Real-time PCR was conducted using 2 *μ*L of cDNA and Power SYBR Green PCR 2 X master mix (Applied Biosystems). Cycling conditions for real-time PCR were as follows: 95°C for 1 min, followed by 40 cycles of 95°C for 15 s and 60°C for 15 s. Real-time PCR was conducted using the Step One Plus Real-time PCR system, and the data were analyzed with StepOne software v2.1 (Applied Biosystems).

### Neuraminidase (recombinant influenza A virus, rvH1N1) inhibition assay

The neuraminidase (NA) inhibition assay was conducted using recombinant NA deduced from the 1918 Spanish flu virus (A/Bervig_Mission/1/18 [H1N1]). NA inhibition activities were determined by Enzyme-Linked Immunosorbent Assay (ELISA). All samples were dissolved in MeOH at 5 mM and diluted. Fifty *μ*L of substrate, 800 *μ*M 4-methylumbelliferyl-α-D-*N*-acetylneuraminic acid sodium salt hydrate solution, was mixed with 80 *μ*L of 50 mM Tris buffer (containing 5 mM CaCl_2 _and 200 mM NaCl, pH 7.5) at room temperature. Twenty *μ*L of the sample solution and 50 *μ*L of NA (0.05 pg/mL in the same Tris buffer) were added to a well in a plate. The mixture was recorded at excitation and emission wavelengths of 365 nm and 445 nm. The inhibition ratio was obtained using the equation:

$$\text{Activity}(\%)=[(S-S_{0})/(C-C_{0}]\times 100$$

Where *C *is the fluorescence of the control (enzyme, buffer, and substrate) after 20 min of incubation, *C*_*0 *_is the fluorescence of the control at zero time, *S *is the fluorescence of the tested samples (enzyme, sample solution, and substrate) after incubation, and *S*_*0 *_is the fluorescence of the tested samples at zero time. To allow for the quenching effect of the samples, the sample solution was added to the reaction mixture *C*, and any reductions in fluorescence were assessed.

## Results

### Cytotoxicity of *Alpinia katsumadai *extracts in MDCK cells

The cytotoxicity of two AK extracts and five AK fractions was evaluated by the MTT assay for 50% cytotoxic concentration (CC_50_). Confluent MDCK cells were incubated with EMEM media in the absence or presence of two-fold diluted AK extracts (0.39-200 *μ*g/mL) for 72 h, and the MTT reagents were treated onto the cells. CC_50 _values of AK-1, AK-2, and AK-3 showed 8.9-27.1 *μ*g/mL. CC_50 _values of AK-5, AK-9, AK-10, and AK-11 showed 92.3-over 200 *μ*g/mL (Table 1). Hence, experiments to evaluate the antiviral effect were carried out at AK extracts and AK fractions concentration below CC_50 _in this study.

**Table 1:** Anti-influeza virus effects of AK extracts and AK fractions by simultaneous treatment assay.

Extract or Compound	CC_50 _(*μ*g/mL)^a^	A/PR/8/34 (H1N1)		A/Chicken/Korea/MS96/96 (H9N2)	
			
		EC_50 _(*μ*g/mL)^b^	SI^c^	EC_50 _(*μ*g/mL)^b^	SI^c^
Tamiflu	>200	<1.5	>133.3	3.5 ± 1.4	57.1
EtOH extract (AK-1)	27.1 ± 0.4	2.6 ± 1.2	10.4	0.6 ± 1.5	45.2
EtOAc fraction (AK-2)	8.9 ± 2.3	3.3 ± 2.3	2.3	1.2 ± 1.9	7.4
H_2_O fraction (AK-3)	21.8 ± 4.2	0.8 ± 1.4	27.3	< 0.39 ± 0.4	>55.9
40% methanol fraction (AK-5)	>200	2.0 ± 3.2	>100	2.1 ± 2.3	> 95.2
H_2_O extract (AK-9)	92.3 ± 4.6	-	-	1.4 ± 0.6	65.9
Polysaccharide fraction (AK-10)	163.5 ± 5.3	12.5 ± 1.1	13.1	2.3 ± 3.6	71.1
Supernatant fraction (AK-11)	119.1 ± 2.4	16.4 ± 4.5	7.3	1.6 ± 0.8	9.3

^a^CC_50 _: mean (50%) value of cytotoxic concentration.

^b^EC_50 _: mean (50%) value of effective concentration.

^c^SI: selective index, CC_50_/EC_50_.

### Inhibitory activity of *Alpinia katsumadai *on influenza virus binding to cell receptors

**Figure 2:**
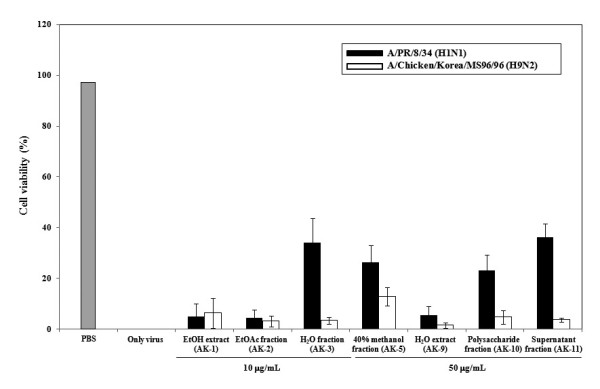
**Antiviral activity of AK extracts and AK fractions before virus attachment in pre-treatment assay**. MDCK cells were pre-incubated with AK extracts and AK fractions 12 h prior to infection of influenza virus (A/PR/8/34 [H1N1] and A/Chicken/Korea/MS96/96 [H9N2]). Antiviral effects were determined by formation of cytopathic effect and plotted as a percentage of cell control (uninfected) and virus control (untreated). AK-1: EtOH extract; AK-2: EtOAc fraction; AK-3: H_2_O fraction; AK-5: 40% methanol fraction; AK-9: H_2_O extract; AK-10: Polysaccharide fraction; AK-11: Supernatant fraction.

In order to test the ability of the two AK extracts and five AK fractions in preventing the attachment of influenza virus to MDCK cells, we used and analyzed the pre-treatment and simultaneous treatment assays. In the pre-treatment assay, AK extracts and AK fractions were treated the MDCK cells and incubated for 12 h before virus infection. Cells were infected with virus and incubated for 72 h at 35°C under 5% CO_2 _atmosphere. The results showed that antiviral effects against A/PR/8/34 (H1N1) and A/Chicken/Korea/MS96/96 (H9N2) were less than 50% inhibition in pre-treatment assay. (Figure 2). In the simultaneous treatment assay, after various concentrations of AK extracts or AK fractions and A/PR/8/34 (H1N1) or A/Chicken/Korea/MS96/96 (H9N2) were mixed, and incubated at 4 °C for 1 h. Confluent MDCK cells were inoculated with the mixture for 1 h. Then, the mixture removed and cells replced with media and were incubated 72 h at 35 °C under 5% CO_2 _atmosphere. We found that AK-1 to AK-3 (EtOH extract, EtOAc fraction, H_2_O fraction), AK-5 (40% methanol fraction), AK-10 (polysaccharide fraction), and AK-11 (supernatant fraction) exhibited inhibitory activities against A/PR/8/34 (H1N1) with EC_50 _values ranging from 0.8 ± 1.4 to 16.4 ± 4.5 *μ*g/mL (Table 1). Against A/Chicken/Korea/MS96/96 (H9N2), the two AK extracts and five AK fractions (AK-1 to AK-3, AK-5, and AK-9 to AK-11) inhibited virus infection with EC_50 _values ranging from <0.39 ± 0.4 to 2.3 ± 3.6 *μ*g/mL (Table 1). One AK extract and five AK fractions (AK-1 to AK-3, AK-5, AK-10, and AK-11) inhibited both A/PR/8/34 (H1N1) and A/Chicken/Korea/MS96/96 (H9N2) influenza virus infection in MDCK cells. Interestingly, A/Chicken/Korea/MS96/96 (H9N2) strain was inhibited strongly demonstrating SI values of 45.2, 55.9, > 95.2 and 71.1 in A/Chicken/Korea/MS96/96 (H9N2) by AK-1, AK-3, AK-5 and AK-10 extracts, respectively (Table 1). Significantly, AK-5 showed the best inhibitory effect in the both A/PR/8/34 (H1N1) and A/Chicken/Korea/MS96/96 (H9N2).

### Hemagglutination inhibition activity

**Figure 3:**
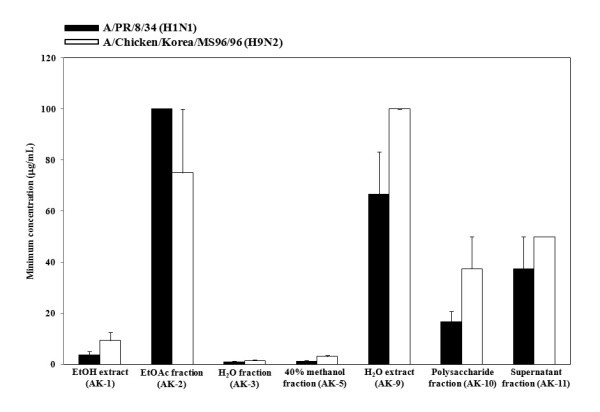
**Inhibitory activity of AK extracts and AK fractions on agglutination with viral hemagglutinin and chichen RBC (cRBC)**. Four HAU of influenza virus (A/PR/8/34 [H1N1] and A/Chicken/Korea/MS96/96 [H9N2]) were mixed with an equal volume of 2-fold diluted two AK extracts and five AK fractions or PBS (negative control) and incubated for 1 h at 4°C. The hemagglutination activity was tested by incubation with 1% cRBC for 1 h at room temperature. We determined the minimum concentration of AK extracts and AK fractions inhibiting the viral hemagglutination completely.

The simultaneous treatment assay results indicated that treatment with AK extracts and AK fractions on virus entry completely abrogated virus infectivity. Hence, we evaluated whether AK extracts inhibit hemagglutination by influenza virus. The two AK extracts and five AK fractions completely inhibited viral attachment onto cRBCs in both A/PR/8/34 (H1N1) and A/Chicken/Korea/MS96/96 (H9N2) at less than 100 *μ*g/mL (Figure 3). The two AK extracts and five AK fractions showed a decreasing order of HI activity, AK-3 (1.0 ± 0.3 *μ*g/mL) > AK-5 (1.3 ± 0.3 *μ*g/mL) > AK-1 (3.6 ± 1.4 *μ*g/mL) > AK-10 (16.7 ± 4.2 *μ*g/mL) > AK-11 (37.5 ± 12.5 *μ*g/mL) > AK-9 (66.7 ± 16.7 *μ*g/mL) > AK-2 (100 ± 0 *μ*g/mL) against A/PR/8/34 (H1N1) and AK-3 (1.6 ± 0.3 *μ*g/mL) > AK-5 (3.1 ± 0.5 *μ*g/mL) > AK-1 (9.4 ± 3.1 *μ*g/mL) > AK-10 (37.5 ± 12.5 *μ*g/mL) > AK-11 (50 ± 0 *μ*g/mL) > AK-2 (75 ± 25 *μ*g/mL) > AK-9 (100 ± 0 *μ*g/mL) against A/Chicken/Korea/MS96/96 (H9N2). Among them, AK-1, AK-3, and AK-5 particularly showed strong inhibition of HA with 1.04 ± 0.3 to 3.64 ± 1.4 *μ*g/mL against A/PR/8/34 (H1N1) and 1.56 ± 0.3 to 9.37 ± 3.1 *μ*g/mL against A/Chicken/Korea/MS96/96 (H9N2). Results demonstrated strong interaction of AK extracts and AK fractions with hemagglutinin on the outer-layer proteins of influenza virus causing the blockage of viral attachment.

### Inhibitory activity of *Alpinia katsumadai *on influenza virus replication

**Figure 4:**
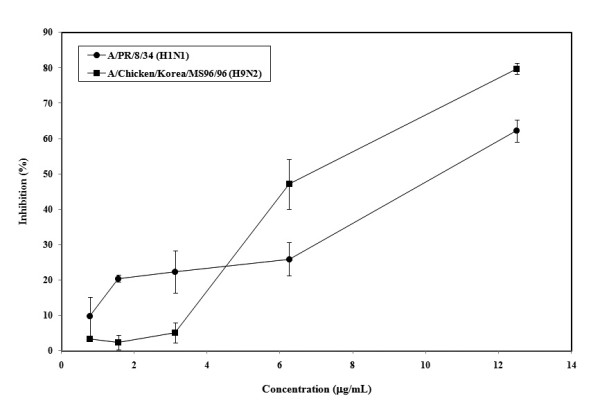
**Antiviral effect of AK-3 after virus entry in post treatment assay**. Influenza viruses at 100 TCID_50 _were inoculated in MDCK cells. After 1 h, viruses were removed and MDCK cells were treated with AK-3 at different concentration. The cultures were incubated for 72 h at 35°C under 5% CO_2 _atmosphere. Each concentration of AK-3 was assayed by two times in triplicate.

The post treatment assay was performed to evaluate whether the two AK extracts and five AK fractions are able to inhibit replication of influenza virus A/PR/8/34 (H1N1) and A/Chicken/Korea/MS96/96 (H9N2) in MDCK cells. Two AK extracts and three AK fractions except AK-3 were showed no inhibitory effects against influenza viruses in the post treatment assay. However, AK-3 demonstrated a dose dependant antiviral activity against A/PR/8/34 (H1N1), and an effective antiviral activity against A/Chicken/Korea/MS96/96 (H9N2) at concentration below 12.5 *μ*g/mL (Figure 4). Similarly in simultaneous assay, AK-3 showed a stronger antiviral effect against A/Chicken/Korea/MS96/96 (H9N2) than against A/PR/8/34 (H1N1).

**Figure 5:**
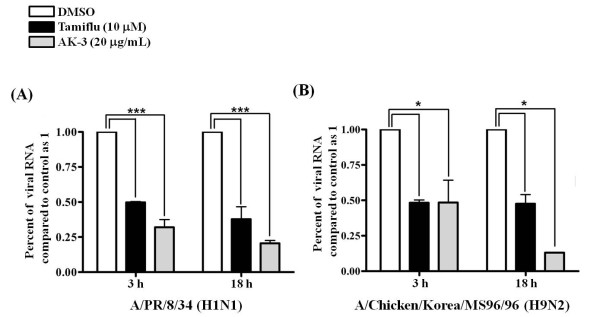
**Quantitive real-time PCR of influenza viral RNA levels normalized to GAPDH**. MDCK cells were infected with 0.01 MOI influenza viruses. After 1 h, viruses were removed. MDCK cells were treated with DMSO (0.5%), AK-3 (20 *μ*g/mL) and Tamiflu (10 *μ*M). Total RNA extraction was performed at 3 h and 18 h after influenza virus infection and the levels of intracellular influenza viral RNA were measured. Influenza viral RNA levles normalized to GAPDH. (A) A/PR/8/34 (H1N1) (*** *p < 0.01*), (B) A/Chicken/Korea/MS96/96 (H9N2) (* *p < 0.05*).

As influenza viral RNA synthesized in early and late-stage, their syntheses were compared between drug-treated (AK-3) and untreated infected cells. RNA extraction was performed at 3 h and 18 h after influenza virus infection and the levels of intracellular influenza RNA were measured. Quantitative real-time PCR showed a reduction of influenza RNA from the AK-3 (20 *μ*g/mL) treated cells comparison with the non-treated cells (05.% DMSO) in both A/PR/8/34 (H1N1) and A/Chicken/Korea/MS96/96 (H9N2) (Figure 5). Specially, influenza viral RNA inhibition of AK-3 (20 *μ*g/mL) treated cells showed a stronger in late stage than in early stage. These results indicate that AK-3 exerts antiviral effects by two mechanisms, blockage of viral attachment and virus replication.

### Neuraminidase inhibition activity

The biological activities of the two AK extracts and five AK fractions were assessed against NAs from recombinant influenza virus A (rvH1N1). We found that the IC_50 _values of the two AK extracts and four AK fractions except AK-11 ranged from 13.2 to 153.1 *μ*g/mL against rvH1N1 NA (Table 2) and these were dose-dependent, respectively.

**Table 2:** Inhibitory effects of AK extracts and AK fractions on neuraminidase from recombinant influenza virus A (rvH1N1).

Extracts and layers	IC_50 _(μg/mL) ^a^
EtOH extract (AK-1)	153.1 ± 22.1
EtOAc fraction (AK-2)	13.2 ± 2.0
H_2_O fraction (AK-3)	25.0 ± 4.1
40% methanol fraction (AK-5)	13.8 ± 2.4
H_2_O extract (AK-9)	20.4 ± 0.7
Polysaccharide fraction (AK-10)	62.6 ± 4.2
Supernatant fraction (AK-11)	-

^a ^All extracts and fractions were examined in a set of duplicated experiment; IC_50 _values of extracts and fractions represent the concentration that caused 50% enzyme activity loss.

## Discussion

The influenza virus replication cycle can be divided into 5 steps: 1) binding of viral HA to sialic acid (SA) receptor on host cell surface (adsorption step), 2) internalization of virus by receptor-mediated endocytosis and fusion of viral HA2 with endosomal membranes triggered by influx of protons through M2 channel (endocytosis and fusion step), 3) release of viral genes into the cytoplasm (uncoating step), 4) packaging of viral proteins with viral genes after viral RNA replication, transcription and translation, and budding of new viruses (packaging and budding step), and 5) release of new viruses by sialidase cleaving SA receptors (release step) [1,18]. Recently, antiviral activity of AK has been reported to inhibit the release of new viruses via putative interaction in the NA binding site on human influenza virus A/PR/8/34 of subtype H1N1 and four H1N1 swine influenza viruses [16]. However, this information is still limited to elucidate the antiviral mechanisms of AK. Therefore, it is hypothesized that the antiviral effect of AK extracts and AK fractions works in two ways: 1) blockage of viral binding to cell receptor, and 2) inhibition of viral replication after entry. To determine the stage at which AK extracts and AK fractions exhibit inhibitory activities, time-of-addition assays at three distinct time points: 12 h prior to infection (pre-treatment), time after incubation and before virus infection (simultaneous treatment), and 1 h after virus entry (post treatment), were performed.

The time-of-addition assays during the pre-treatment and simultaneous treatment were used to identify which extracts block the viral adsorption to cells. The pre-treatment assay did not show significant antiviral activity (Figure 2). AK-3 and AK-11 showed a weak inhibitory effect of A/PR/8/34 (H1N1) only. In simultaneous treatment assay, two AK extracts and five AK fractions completely abrogated virus infectivity by lowering the concentration of both A/PR/8/34 (H1N1) and A/Chicken/Korea/MS96/96 (H9N2). AK-9 (H_2_O extract) showed antiviral effect against A/Chicken/Korea/MS96/96 (H9N2) only. These data suggest that two AK extracts and five AK fractions may directly interfere with viral envelope protein and not with the SA receptor at the cell surface. Therefore, we used HI assays to determine whether the two AK extracts and five AK fractions interacted with HA of influenza virus. Two AK extracts and five AK fractions exhibited complete inhibition of viral HA in both A/PR/8/34 (H1N1) and A/Chicken/Korea/MS96/96 (H9N2), which agrees with the simultaneous treatment assay results. Overall, we strongly suggest that AK extracts and AK fractions could develop potent antiviral drug candidate via inhibition of viral HA protein.

To evaluate the anti-influenza activity after virus infection, we employed the post treatment assay and quantitative real-time RCR to test the *in vitro *effect of these extracts and fractions on viral replication. Grienke et al.[16] reported about the AK fractions of dichloromethane, ethyl acetate, and *n*-butanol inhibited NA protein. Therefore, we evaluated NA inhibition assay and confirmed that two AK extracts and four AK fractions except AK-11 inhibited NA protein of rvH1N1. We found that only AK-3 inhibited influenza virus infection suggesting two possible ways of viral inhibitions, 1) blockage of viral attachment by inhibition of viral HA protein, 2) blockage of viral replication and/or release by inhibition of NA. Interestingly, AK-3 showed greater inhibition of viral attachment than of replication, against both A/PR/8/34 (H1N1) and A/Chicken/Korea/MS96/96 (H9N2). However, the other two AK extracts and four AK fractions showed only blockage of viral attachment of influenza virus to the cell.

## Conclusions

This study has shown that AK extracts and AK fractions can inhibit both A/PR/8/34 (H1N1) and A/Chicken/Korea/MS96/96 (H9N2) influenza viruses by inhibiting viral HA binding to the SA receptors in the host cell. AK-3 offers a potential antiviral drug candidate by inhibiting viral attachment step, replication and release step. These results lead to further investigation about characterization of active compounds and their specific mechanism against influenza virus.

## Competing interests

The authors declare that they have no competing interests.

## Authors' contributions

HJK and HHK carried out most of the experiments, analyzed the data and participated in writing. SYY and JSC cellular studies and drafted the manuscript. YBR prepared extractions and fractions. KOC and MCR contributed to the data analysis. SJP and WSL conceived the study, participated in its design and coordination and accounted for the manuscript writing. All authors read and approved the final manuscript.
